# Higher-Order Conditioning With Simultaneous and Backward Conditioned Stimulus: Implications for Models of Pavlovian Conditioning

**DOI:** 10.3389/fnbeh.2021.749517

**Published:** 2021-11-11

**Authors:** Arthur Prével, Ruth M. Krebs

**Affiliations:** Department of Experimental Psychology, Ghent University, Ghent, Belgium

**Keywords:** backward conditioning, higher-order conditioning, reinforcement learning, reward prediction error, simultaneous conditioning

## Abstract

In a new environment, humans and animals can detect and learn that cues predict meaningful outcomes, and use this information to adapt their responses. This process is termed Pavlovian conditioning. Pavlovian conditioning is also observed for stimuli that predict outcome-associated cues; a second type of conditioning is termed higher-order Pavlovian conditioning. In this review, we will focus on higher-order conditioning studies with simultaneous and backward conditioned stimuli. We will examine how the results from these experiments pose a challenge to models of Pavlovian conditioning like the Temporal Difference (TD) models, in which learning is mainly driven by reward prediction errors. Contrasting with this view, the results suggest that humans and animals can form complex representations of the (temporal) structure of the task, and use this information to guide behavior, which seems consistent with model-based reinforcement learning. Future investigations involving these procedures could result in important new insights on the mechanisms that underlie Pavlovian conditioning.

## Introduction

**Figure 1:**
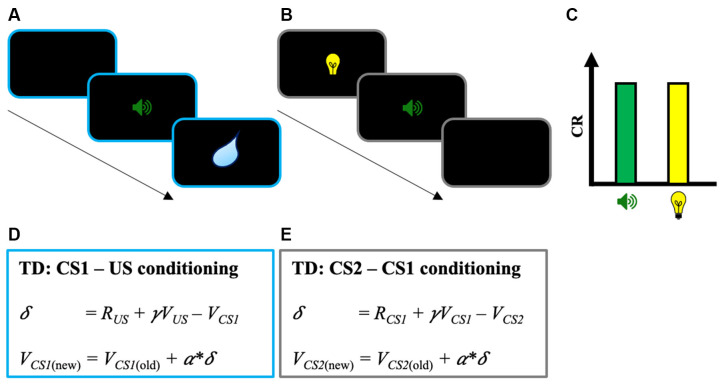
Illustration of the second-order conditioning procedure. **(A)** Phase 1: First-order conditioning between a stimulus (CS1—sound) paired with an unconditioned stimulus (US—water). **(B)** Phase 2: Second-order conditioning between a second stimulus (CS2—light) paired with the previously paired stimulus CS1. **(C)** Classic results found in the second-order conditioning task with the conditioned response (CR) evoked both by CS1 and CS2. In sensory preconditioning, the procedure is similar except that phases 1 and 2 are inversed. **(D)** TD learning for the first-order conditioning phase with change in CS1’s predicted value *V*_CS1_. Note that *V*_US_ is zero because of the absence of predicted value at the time of the US. Because *R*_US_ is positive, the pairing between CS1 and the US results in a positive *δ* (i.e., *R*_US_ − V_CS1_ > 0), and the acquisition of predicted value from CS1 through the update of *V*_CS1_ (*V_CS1_*_(new)_ = *V_CS1_*_(old)_ + α*δ). **(E)** TD learning for the second-order conditioning phase with change in CS2’s predicted value *V_CS2_*. Note that *R_CS1_* is zero because of the absence of reward at CS1. Here, the positive *V_CS1_* learned during the first-order conditioning phase is sufficient to produce a positive *δ* (i.e., γ *V*_CS1_ − *V*_CS2_ > 0) and to increase the predicted value from CS2 (*V_CS2_*). TD, Temporal Difference.

When being exposed to a new environment, humans and other animals can detect and learn that cues or contextual stimuli predict the prospect of meaningful events. This learning process and the behavioral change associated are classically named Pavlovian conditioning (Hollis, 1997; Fanselow and Wassum, 2015). Not limited to the pairing between a stimulus and an outcome, Pavlovian conditioning is also observed for stimuli that predict outcome-associated cues. This second type of conditioning, in which a cue predicts another predictive stimulus, is referred to as higher-order Pavlovian conditioning (Gewirtz and Davis, 2000). Higher-order conditioning is particularly interesting as it is an excellent way to understand how humans and other animals form complex representations of the structure of the environment, and how they use these representations to guide flexible responses (Jones et al., 2012; Sadacca et al., 2016, 2018; Wang et al., 2020; Chandran and Thorwart, 2021). In the lab, higher-order conditioning is studied by second-order conditioning or sensory preconditioning (e.g., Gewirtz and Davis, 2000; Parkes and Westbrook, 2011). In second-order conditioning, a stimulus (CS1) is first paired with an unconditioned stimulus (US) until CS1 evokes a conditioned response (CR). Then, in a subsequent phase, a second stimulus (CS2) is paired with CS1 but without the US. At the end of the second phase, and despite the absence of direct pairing with the US, the presentation of CS2 alone is sufficient to evoke a CR (see Figures 1A–C; Rizley and Rescorla, 1972; Rashotte et al., 1977). The pairing procedure used in sensory preconditioning is similar to second-order conditioning except that the order of phases 1 and 2 is inversed (i.e., CS2 → CS1 pairings, then CS1 → US pairings; Rescorla and Cunningham, 1978).

Traditionally, investigations on higher-order conditioning involve forward CS2 → CS1 and CS1 → US pairings. However, far less investigated are procedures involving simultaneous or backward pairings (e.g., Prével et al., 2019). In this mini-review, we will argue that these procedures are actually particularly relevant for the understanding of Pavlovian conditioning. Results from these experiments are indeed difficult to interpret in terms of the Reward Prediction Error (RPE) hypothesis (Schultz and Dickinson, 2000) and for models that implement this learning-rule like Temporal Difference (TD) learning models (Sutton and Barto, 2018). On the opposite end, the results seem to be conceptually consistent with model-based reinforcement learning systems (Daw et al., 2005; Gläscher et al., 2010; O’Doherty et al., 2017) and call for new investigations on the underlying computational mechanisms. After a presentation of the RPE hypothesis and a description of how a TD approach can account for higher-order conditioning, we will present results from higher-order conditioning studies that used simultaneous and backward pairing. We will discuss how far they are difficult to interpret from a reward prediction error perspective and how they seem to support model-based reinforcement learning systems. We will conclude this mini-review by discussing the perspectives offered by follow-up studies on higher-order conditioning with simultaneous and backward pairing.

## Reward Prediction Error and Higher-Order Conditioning

Historically, one of the most dominant hypotheses about Pavlovian acquisition has been the RPE hypothesis (Schultz and Dickinson, 2000; Niv and Schoenbaum, 2008). This hypothesis states that a change in the value of a CS is driven by the discrepancy between the outcome expected from that stimulus, and the outcome actually received. Quantitative formulations of the RPE hypothesis are now largely based on TD learning (Niv and Schoenbaum, 2008; Ludvig et al., 2012; Sutton and Barto, 2018). Close to the Rescorla and Wagner (1972) model in terms of learning rule, TD models present the advantage of solving some of its important failures. The TD approach makes notably successful predictions about second-order conditioning, a phenomenon difficult to explain in terms of the Rescorla and Wagner model (Miller et al., 1995). In TD models, RPE (*δ*) is defined by:


$$\delta_{t+1}\;=\;R_{t+1}\;+\;\gamma V_{t+1}\;-\;V_{t}$$

Where *R*_*t*+1_ is the observed reward at *t*+1, *V_t_*_+1_ and *V_t_* are the predicted value at *t*+1 and *t*, and *γ* is a discount factor (with 0 < *γ* ≤ 1). δ is used to update the prediction made at *t* by:


$$V_{t}\;=\;V_{t}\;+\;\alpha \;(\delta_{t+1})$$

Where α is a learning rate parameter (with 0 < α ≤ 1).

Using this learning rule the TD models of Pavlovian conditioning can successfully explain second-order conditioning (Seymour et al., 2004; Sutton and Barto, 2018; Maes et al., 2020; see Figures 1D,E). In the first phase of the procedure, the pairing between CS1 and the US results in a positive δ and the acquisition of predicted value from CS1 (i.e., positive *V*_CS1_). Then, this predicted value can be used to drive learning on CS2 in the second phase of the procedure. Despite the absence of reward during the second-order conditioning phase (i.e., *R*_CS1_ = 0), the positive value of *V*_CS1_ is sufficient to produce a positive δ (i.e., γ *V*_CS1_ − *V*_CS2_ > 0) and to increase the predicted value from CS2 (*V*_CS2_). Interestingly, at the neural level, it has been found that the activity of dopaminergic neurons in a similar task moves backward from the US to the first predictive stimulus cue (i.e., CS2), as it would be predicted by TD models (Schultz, 2015).

Thus, TD learning seems particularly relevant to understanding the acquisition of higher-order predictive values, both at a behavioral and a neural level. The approach, however, is not without limitations. Particularly, the model fails to explain the acquisition of predictive value by CS2 in sensory preconditioning tasks: Due to the absence of reward in phase 1 and the predicted value of zero for CS1 (i.e., *R*_CS1_ + γ *V*_CS1_ = 0), a change in *V*_CS2_ is not expected according to TD models. However, the evidence from measuring responses to CS2 suggests the acquisition of predicted value from the stimulus. This challenge to TD learning has been repeatedly highlighted in the literature, and it becomes one of the arguments against the hypothesis that Pavlovian conditioning is only driven by RPE (Niv and Schoenbaum, 2008; Sadacca et al., 2016). Much less considered is the challenge posed by results from higher-order conditioning studies that involve a simultaneous or a backward CS1. Here, we believe that these results are particularly relevant for our understanding of higher-order learning. The next section will be dedicated to these findings.

## Higher-Order Conditioning with Simultaneous and Backward Pairing

**Figure 2:**
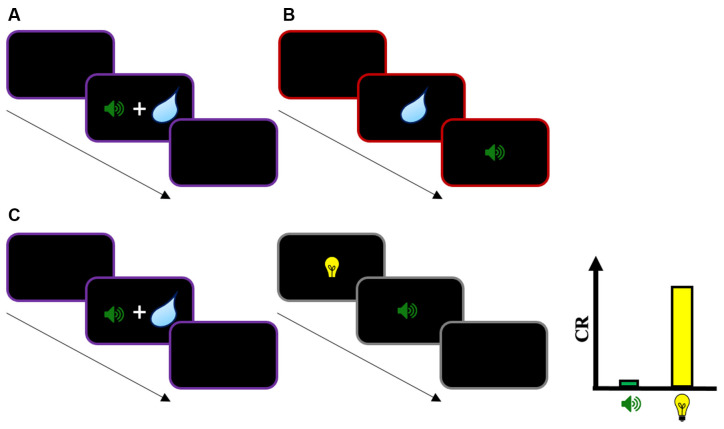
Illustration of simultaneous, backward, and second-order conditioning with simultaneous CS1. **(A)** Simultaneous conditioning with a stimulus (CS1—sound) presented simultaneously with an unconditioned stimulus (US—water). **(B)** Backward conditioning with CS1 presented after a US. **(C)** Second-order conditioning with simultaneous CS1: In phase 1, a stimulus CS1 is presented simultaneously with a US. In phase 2, a second stimulus CS2 is paired with CS1 through forward pairing. During the test, while CS1 will evoke low conditioned response (CR), CS2 will evoke substantial CR. According to the TD account, a low CR evoked by CS1 is expected because CS1 is not followed by the US (i.e., *R*_US_ = 0) in phase 1. In addition, a change in CS2 value (*V*_CS2_) depends directly on CS1’s own value (*V*_CS1_). Thus, a second-order pairing with a first-order stimulus CS1 that evokes a low CR level (and with presumably a low predicted value) should result in low responding to CS2. The evidence of substantial response to that stimulus challenges the TD account. The same holds for a model-based account of higher-order learning if the change in *V*_CS2_ depends on CS1’s own predicted value *V*_CS1_. Instead, it seems necessary for CS2’s predicted value to be based on US expectations to account for this finding. Note that the same pattern of results is observed for second-order conditioning with backward CS1, and for sensory preconditioning with simultaneous and backward CS1.

In Pavlovian conditioning, the classic pairing procedure used to study the acquisition of new stimulus-outcome associations is the forward procedure in which the CS precedes the presentation of the US. Contrasting with this, in simultaneous and backward pairing the CS is presented simultaneously and after the US, respectively (Figures 2A,B). Experiments that used these procedures classically showed low response rates to the CS, or even the development of conditioned inhibition (Spooner and Kellogg, 1947; Fitzwater and Reisman, 1952; Moscovitch and LoLordo, 1968; Siegel and Domjan, 1974; but see Spetch et al., 1981; Prével et al., 2016). These observations suggest that simultaneous and backward pairings are not appropriate procedures to produce a robust CR, which is consistent with TD models: When a simultaneous or a backward CS is presented, the stimulus is never followed by a reward at *t*+1. Thus, a change in *V*_CS_ is consequently not expected from those pairing procedures. In addition, a higher-order cue (CS2) that precedes a simultaneous or a backward CS1 should not produce robust responding because *V*_CS1_ is zero at the end of the first-order conditioning phase.

From a functional perspective, the absence of a robust CR in simultaneous and backward pairing is not surprising if we consider that the function of the response is to prepare the organism for the US (Hollis, 1997). Because the CS is not predictive of the US, there is a priori no reason to expect a preparatory response evoked by that stimulus. However, what is not clear is whether the absence of a CR measured to the simultaneous or backward CS really means that subjects did not learn anything from these pairing procedures due to the RPE of zero. Alternatively, it is possible that subjects in these experiments learned an association between the simultaneous or backward CS and the US, but these associations are simply not overtly expressed due to the absence of predictive value of the CS (Arcediano and Miller, 2002). In what follows, we will discuss the results from higher-order conditioning studies that support this interpretation.

For example, Barnet et al. (1991) tested whether a first-order stimulus CS1 paired simultaneously with a US can support the conditioning of a second-order stimulus CS2 (see Figure 2C for an illustration). Consistent with common findings in simultaneous conditioning studies, the authors reported low responses evoked by CS1 in comparison to a forward first-order stimulus, supporting the idea that the procedure is not efficient to produce a robust CR. However, when in a subsequent phase a second-order stimulus CS2 was paired with CS1 using a forward pairing (i.e., CS2 → CS1 pairings), the authors found a substantial level of CR evoked by CS2, despite the low response measured on CS1. These results by Barnet et al. (1991) seem difficult to explain in terms of TD learning. According to the account described above, a change in CS2 value (*V*_CS2_) depends directly on CS1’s own value (*V*_CS1_). Thus, a second-order pairing with a first-order stimulus CS1 that evokes low response (and with presumably a low predicted value) should result in low response to CS2. However, the evidence of substantial response to that stimulus challenges this interpretation. Later, Barnet and Miller (1996) extended their investigations to backward conditioning. In phase 1 of a second-order conditioning task, a first-order stimulus CS1 was paired to a US using backward pairing. This resulted in the development of conditioned inhibition, a classic result of this pairing procedure. Interestingly, when in phase 2 a second-order stimulus CS2 was paired with CS1 using forward pairing, this resulted in substantial CR to CS2 despite the inhibitory status of CS1. Again, the result is problematic for the TD account of second-order conditioning. It is not clear why a first-order stimulus CS1 with an acquired inhibitory status (and presumably, a negative predicted value *V*_CS1_) can support the conditioning of a second-order stimulus CS2.

These results by Barnet and Miller (1996) were replicated by Cole and Miller (1999), who found that the effect varied with the number of backward pairing trials in phase 1. More exactly, the authors reported that a backward CS1 supports second-order conditioning only when the number of backward pairing trials is low or high, but the CR to CS2 decreases at an intermediate number of trials. Parallel to these investigations, Barnet et al. (1997) demonstrated that a backward CS1 can support stronger second-order conditioning compared to a forward first-order CS1, despite a lower CR to that backward stimulus. More recent observations by Prével et al. (2019) are consistent with these findings. Specifically, the authors demonstrated that a second-order stimulus CS2 can function as an efficient conditioned reinforcer for instrumental response in the test phase, even when that stimulus was paired with a backward CS1 that did not evoke CR during phase 2. Finally, similar findings were reported using sensory preconditioning. For example, Matzel et al. (1988) found evidence of substantial sensory preconditioning with simultaneous and backward first-order paired stimuli. Barnet et al. (1997) reported results similar to their observations in a second-order conditioning task with sensory preconditioning. Finally, Arcediano et al. (2003) found successful sensory preconditioning with backward first-order CS. In summary, it seems clear from all these experiments that a simultaneous or backward first-order CS can support higher-order learning, even if that same stimulus shows a low CR level or conditioned inhibition. As we have seen, the evidence is difficult to explain based on TD models, and particularly with regard to sensory preconditioning due to the additional absence of RPE in phase 1. In the next section, we will describe the model-based reinforcement learning account as a valuable alternative to TD learning.

## Model-Based Reinforcement Learning and Higher-Order Conditioning

Because of the challenges posed by effects like sensory preconditioning, the last 10–20 years have seen the development of another class of models termed model-based reinforcement learning (Daw et al., 2005; Gläscher et al., 2010; O’Doherty et al., 2017). In this approach, human subjects and animals can learn a model of the environment to guide appropriate responding. This model includes the states encountered by the subjects, as well as the transition probabilities between states and the available rewards. This contrasts with (model-free) TD models in which the subjects merely learn the predicted value of each state, but not the potential transition between states. Another characteristic of the model-based approach resides in the fact that the subjects can use the learned-transitions between states to update the states’ value through a (mental) simulation mechanism. This second aspect is particularly interesting because it can be used to account for goal-directed phenomena like devaluation (e.g., Wilson et al., 2014), but certainly also sensory preconditioning: Here, during phase 1 participants would learn the transition probability between CS2 and CS1, before learning during phase 2 the positive predictive value of CS1 based on its direct pairing with the US. Then, through a simulation mechanism, the learned transition between CS2 and CS1 and the expected value from *V*_CS1_ could be used to update *V*_CS2_, i.e., subjects could (mentally) assign a new value to CS2 based on the learned-transition between CS2 and CS1 (i.e., CS2 is followed by CS1), and the learned predicted value from CS1. For example, if we adapt the model-based mechanism proposed by Wilson et al. (2014) to sensory preconditioning, at the end of training the (model-based) value of CS2 could be updated through:


$$V_{CS2}\;=\;V_{CS1}\;\times \;p\;(CS1\;∥\;CS2)$$

Where *p*(CS1∥CS2) is the estimated learned probability of CS2 leading to CS1, and *V*_CS1_ is the predicted value from CS1. Because *p*(CS1∥CS2) and *V*_CS1_ are positive due to the pairings in phases 1 and 2, this would result in a positive *V*_CS2_ and the ability of the stimulus to evoke CR.

In addition to sensory preconditioning, the model-based learning approach seems also very promising to account for the findings presented in the previous section. The assumption that humans and animals can learn a model representing the structure of the environment, and that they use this model to flexibly update the value of states (stimuli) and guide responding, seems remarkably consistent with the results described above. In these experiments, it is as if subjects learned the (temporal) structure of the task and used this structure to infer a predictive value from CS2 and guide responding: Participants first learned that CS1 is presented simultaneously or after the US, but the absence of predictive value of CS1 prevented the development of a robust CR. However, through the integration of the associations learned in phases 1 and 2, the forward pairing between CS2 and CS1 conferred a predictive value between CS2 and (the representation of) the US, which resulted in the CR measured in response to this stimulus (see Arcediano and Miller, 2002). Interestingly, multiple results in the literature suggest the acquisition of such temporal maps (e.g., Cole et al., 1995; Arcediano et al., 2005; Thrailkill and Shahan, 2014). However, it must be noted that it is not clear what the exact computational mechanism is that supports the temporal integration and the acquired predicted value on CS2 observed in these studies. If we consider for example the model-based mechanism described above, because a change in *V*_CS2_ depends in this formulation on CS1’s own predicted value *V*_CS1_, the problem remains that it is difficult to understand why a stimulus that shows low CR or conditioned inhibition supports substantial CR to CS2. Instead, it seems necessary for CS2’s predicted value to be based on US expectation to explain the results presented in the previous section. More investigations will be necessary to propose a complete account of higher-order learning, and particularly a mechanism that allows the temporal integration of the task structure to guide flexible and adaptive responses.

## Conclusion and Outlook

The evidence from sensory preconditioning and higher-order conditioning with simultaneous and backward pairing pose a challenge to the assumption that Pavlovian conditioning is driven only by RPE. Rather, these observations suggest that subjects were able to learn a representation of the (temporal) structure of the task and to use this representation to guide their responses, which seems consistent with the assumptions of model-based reinforcement learning. However, the exact nature of the computational mechanisms is still missing. Here, we are highlighting three fruitful directions for future investigations on higher-order conditioning with simultaneous and backward CS. First, it must be noted that model-free reinforcement learning approaches such as TD models are not necessarily dismissed by these results. To the best of our knowledge, the consensus in the literature seems to assume a co-existence of both model-free and model-based reinforcement learning systems, representing habitual and goal-directed behaviors, respectively (Gläscher et al., 2010; Wilson et al., 2014; O’Doherty et al., 2017). Additional investigations on higher-order conditioning with simultaneous and backward pairing could provide new insights regarding the computational mechanisms that underly model-based reinforcement learning and temporal integration in higher-order conditioning, as well as how model-free and model-based reinforcement learning computations are integrated in that context. Second, an important research question in the study of higher-order conditioning concerns the nature of the associations learned (Gewirtz and Davis, 2000). New investigations using the procedures described in this mini-review could give new insights into what is learned by subjects in these tasks, which in turn could have important implications on the underlying computational mechanisms. Finally, an important hypothesis in the neuroscientific domain is that the phasic activity of dopaminergic neurons represents the RPE teaching signal in the context of model-free reinforcement learning (Schultz, 2015). However, recent results suggest instead that this activity could reflect model-based computations (Sadacca et al., 2016; Sharpe et al., 2017; Langdon et al., 2018; Sharpe and Schoenbaum, 2018). Here, it might be interesting to study how this activity changes during the presentation of CS1 and CS2 depending on the pairing conditions and to test which neural structures are subserving task representations and value updates.

## Author Contributions

Both authors contributed to the article and approved the submitted version.

## Conflict of Interest

The authors declare that the research was conducted in the absence of any commercial or financial relationships that could be construed as a potential conflict of interest.

## Publisher’s Note

All claims expressed in this article are solely those of the authors and do not necessarily represent those of their affiliated organizations, or those of the publisher, the editors and the reviewers. Any product that may be evaluated in this article, or claim that may be made by its manufacturer, is not guaranteed or endorsed by the publisher.
